# Reduction of Mitochondrial Calcium Overload via MKT077-Induced Inhibition of Glucose-Regulated Protein 75 Alleviates Skeletal Muscle Pathology in Dystrophin-Deficient *mdx* Mice

**DOI:** 10.3390/ijms25189892

**Published:** 2024-09-13

**Authors:** Mikhail V. Dubinin, Anastasia E. Stepanova, Irina B. Mikheeva, Anastasia D. Igoshkina, Alena A. Cherepanova, Eugeny Yu. Talanov, Ekaterina I. Khoroshavina, Konstantin N. Belosludtsev

**Affiliations:** 1 Department of Biochemistry, Cell Biology and Microbiology, Mari State University, pl. Lenina 1, Yoshkar-Ola 424001, Russia; lady.stepanowa2010@yandex.ru (A.E.S.); anastasi.igoshkina@yandex.ru (A.D.I.); alyona_2022@bk.ru (A.A.C.); katya_bs@mail.ru (E.I.K.); bekonik@gmail.com (K.N.B.); 2 Laboratory of Mitochondrial Transport, Institute of Theoretical and Experimental Biophysics, Russian Academy of Sciences, Institutskaya 3, Pushchino 142290, Russia; mikheirina@yandex.ru (I.B.M.); evg-talanov@yandex.ru (E.Y.T.)

**Keywords:** Duchenne muscular dystrophy, *mdx* mice, skeletal muscle mitochondria, calcium, MKT077, GRP75, UPR

## Abstract

Duchenne muscular dystrophy is secondarily accompanied by Ca^2+^ excess in muscle fibers. Part of the Ca^2+^ accumulates in the mitochondria, contributing to the development of mitochondrial dysfunction and degeneration of muscles. In this work, we assessed the effect of intraperitoneal administration of rhodacyanine MKT077 (5 mg/kg/day), which is able to suppress glucose-regulated protein 75 (GRP75)-mediated Ca^2+^ transfer from the sarcoplasmic reticulum (SR) to mitochondria, on the Ca^2+^ overload of skeletal muscle mitochondria in dystrophin-deficient *mdx* mice and the concomitant mitochondrial dysfunction contributing to muscle pathology. MKT077 prevented Ca^2+^ overload of quadriceps mitochondria in *mdx* mice, reduced the intensity of oxidative stress, and improved mitochondrial ultrastructure, but had no effect on impaired oxidative phosphorylation. MKT077 eliminated quadriceps calcification and reduced the intensity of muscle fiber degeneration, fibrosis level, and normalized grip strength in *mdx* mice. However, we noted a negative effect of MKT077 on wild-type mice, expressed as a decrease in the efficiency of mitochondrial oxidative phosphorylation, SR stress development, ultrastructural disturbances in the quadriceps, and a reduction in animal endurance in the wire-hanging test. This paper discusses the impact of MKT077 modulation of mitochondrial dysfunction on the development of skeletal muscle pathology in *mdx* mice.

## 1. Introduction

Duchenne muscular dystrophy (DMD) is an X-linked recessive inherited disorder caused by mutations in the *DMD* gene encoding the protein dystrophin [1]. This is the most common hereditary form of muscular dystrophy, diagnosed in an average of 1 in 5000 boys [2]. The severe phenotype of this pathology is primarily due to the lack of production of the full-length isoform of dystrophin Dp427m in skeletal muscles and cardiomyocytes [3]. Dystrophin plays a key role in providing the connection of the cytoskeleton of muscle fibers and cells with the sarcolemma, channel, signaling, and scaffolding proteins and with the extracellular matrix through the dystrophin-associated protein complex, which maintains the structural integrity of muscle tissue and its functional activity [2,4]. The loss of dystrophin in this complex framework structure is accompanied by the progressive destabilization of the muscle fiber membrane and an increase in its permeability to extracellular matrix ions, primarily calcium ions, which is caused by both the appearance of mechanical ruptures of the sarcolemma and dysfunction of ion channels [2,4,5]. It is important to note that calcium overload of muscle fibers is also secondarily caused by the release of large amounts of calcium ions from intracellular depots, primarily from the sarcoplasmic reticulum (SR) [4,5]. All this contributes to calcium overload of muscle fibers, which, in turn, leads to an increase in the activity of Ca^2+^-dependent proteases (calpains) and massive proteolysis of cellular proteins, an increase in the production of reactive oxygen species (ROS) and oxidative stress, the development of chronic inflammation and necrosis, inhibition of regenerative capacity, and fibrosis of muscle tissue [2,4,5].

Energy supply to striated muscles is largely provided by the function of mitochondria [6]. And Ca^2+^ homeostasis in mitochondria is essential for key enzymes in glucose metabolism and respiratory chain complexes [7,8]. However, critically high concentrations of Ca^2+^ contribute to mitochondrial dysfunction. Indeed, it has been previously shown that, in dystrophin-deficient muscles of *mdx* mice [9] or *δ*-sarcoglycan-deficient mice [10], mitochondria are in a state of chronic calcium overload, which contributes to the development of dysfunction of these organelles. This is reflected in a decrease in the intensity of oxidative phosphorylation, ROS overproduction, as well as a dramatic increase in the sensitivity of mitochondria to the induction of the calcium-dependent mitochondrial permeability transition (MPT) pore, a non-selective protein channel in the inner and outer mitochondrial membranes. Pharmacological or genetic desensitization of mitochondria to the opening of the MPT pore, accompanied by an increase in the calcium-buffering capacity of mitochondria, has been shown to significantly improve the functional activity of organelles and normalize their ultrastructure, and also helps to reduce the intensity of destructive processes in dystrophin-deficient skeletal muscles [11,12,13,14,15].

It is generally accepted that the fine regulation of mitochondrial function is mediated by calcium transport from the SR to mitochondria via mitochondria-associated membranes (or MAM contacts) [8,16]. In addition to other proteins, MAM contacts are formed by the voltage-dependent anion channel, which is located on the outer mitochondrial membrane, the inositol 1,4,5-triphosphate receptor of the sarcoplasmic reticulum and their linker glucose-regulated protein 75 (GRP75) [8,16]. Recent data demonstrate that GRP75 directly regulates Ca^2+^ transfer from the ER/SR to mitochondria [17] and support an important role of this protein in the development of mitochondrial dysfunction in diabetes retinopathy [18], nephropathy [19], neurological diseases [20] and myocardial infarction [21]. It has been previously shown that rhodacyanine dye MKT077, a GRP75-specific inhibitor, which is able to bind to GRP75 and abrogate its activity [22,23], prevents the calcium overload of nerve cell mitochondria in the oxygen and glucose deprivation model, leading to improved mitochondrial structure and function [23].

In this work, we assessed the effect of MKT077 administration on calcium overload of skeletal muscle mitochondria in dystrophin-deficient *mdx* mice and concomitant mitochondrial dysfunction, contributing to the development of skeletal muscle pathology. We have shown that daily intraperitoneal administration of this agent at a concentration of 5 mg/kg for 28 days helps to reduce the calcium overload of mitochondria in the skeletal muscles of *mdx* mice, reduce the intensity of oxidative stress in these organelles, and also alleviate the intensity of calcification, degeneration, and fibrosis in skeletal muscles of model animals.

## 2. Results

### 2.1. MKT077 Reduces Calcium Overload and Oxidative Stress in Skeletal Muscle Mitochondria of mdx Mice but Has No Effect on Impaired Oxidative Phosphorylation and Reduced ATP Levels

**Figure 1:**
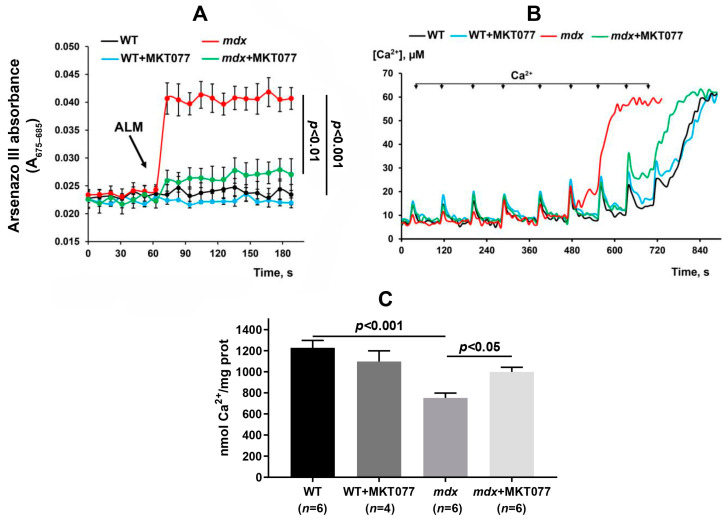
Effect of MKT077 on mitochondrial calcium homeostasis. (**A**) Mitochondrial Ca^2+^ load assay; 0.1 mg/mL alamethicin (ALM) was added to induce maximal calcium release from the mitochondrial matrix. The results are presented as means ± SEM (*n* = 4). (**B**) Changes in the external [Ca^2+^] upon the successive addition of 20 μM Ca^2+^ pulses to the suspension of the skeletal muscle mitochondria of the experimental animals. (**C**) Calcium retention capacity of the skeletal muscle mitochondria. The results are presented as means ± SEM.

It is known that excessive accumulation of calcium ions in dystrophin-deficient muscle fibers is also accompanied by calcium overload of mitochondria, which contributes to organelle dysfunction [9]. Indeed, Figure 1A shows that the application of the permeabilizing agent alamethicin to mitochondria isolated from the quadriceps of *mdx* mice results in a marked increase in the absorption of the calcium-sensitive probe arsenazo III, indicating the release of calcium ions from the mitochondrial matrix. At the same time, calcium release is not observed in the case of the skeletal muscle mitochondria of wild-type mice. This indicates that skeletal muscle mitochondria from dystrophin-deficient mice are in a state of chronic calcium overload. It can be seen that mitochondria isolated from the skeletal muscles of *mdx* mice treated with MKT077 are characterized by a decrease in the amount of calcium ions released from the organelle matrix, which indicates a decrease in the calcium overload of mitochondria. Moreover, MKT077 increases the ability of skeletal muscle mitochondria of *mdx* mice to absorb external calcium ions, which is reflected in an increase in the calcium retention capacity parameter to the level of wild-type animals (Figure 1B,C). One should note that MKT077 had no effect on the calcium level and calcium retention capacity of skeletal muscle mitochondria in wild-type mice.

**Figure 2:**
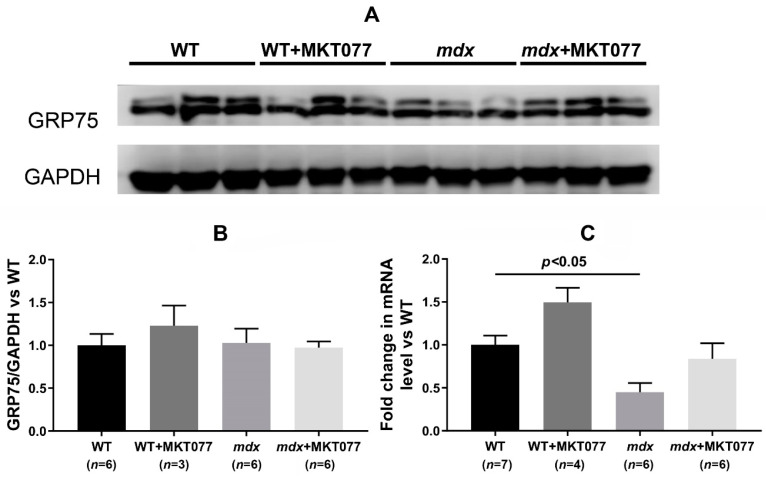
Western blotting of GRP75 and GAPDH (**A**), quantification of GRP75/GAPDH ratio (**B**) and mRNA expression of *Hspa9* relative to *Rplp2* (**C**) in the skeletal muscles of experimental groups of mice. The results are presented as means ± SEM.

The specific target of MKT077 is known to be the GRP75 protein, which controls calcium transport into mitochondria [17]. One can see that the skeletal muscles of *mdx* and wild-type mice do not differ in GRP75 protein levels, despite a slight decrease in the expression level of the *Hspa9* gene encoding this protein (Figure 2). MKT077 has no effect on either the content of this protein in the skeletal muscles of both groups of animals or the level of HSPA9 mRNA (Figure 2). Thus, the effect of MKT077 on mitochondrial calcium homeostasis is not accompanied by changes in the level of this key modulator of calcium transport between the SR and mitochondria.

**Figure 3:**
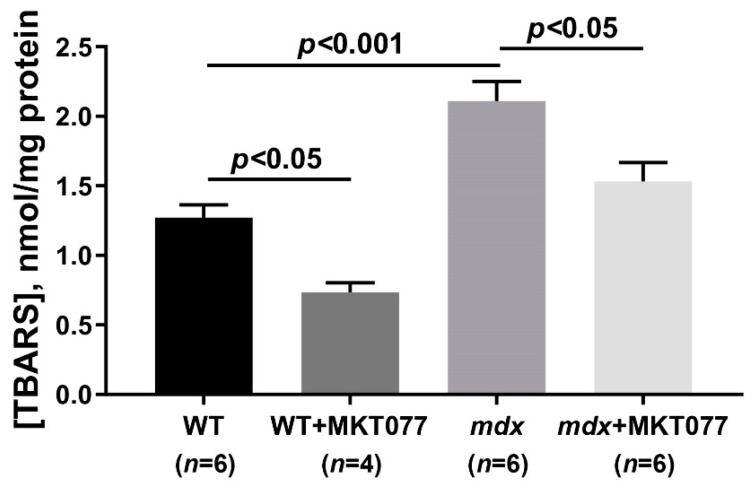
Effect of MKT077 on lipid peroxidation in mitochondria. Lipid peroxidation was assessed by the level of TBARS in the skeletal muscle mitochondria of the experimental animals. The results are presented as means ± SEM.

Excess calcium ions in the mitochondrial matrix cause the overproduction of reactive oxygen species [8]. The level of ROS, as well as their products, increases significantly in dystrophin-deficient muscles, including in mitochondria [9,11,24]. Indeed, the skeletal muscle mitochondria of *mdx* mice show a 1.8-fold increase in thiobarbituric reactive substances (TBARS, mainly represented by malondialdehyde, MDA) compared with wild-type animals, indicating an intensification of mitochondrial membrane lipid peroxidation (Figure 3). MKT077 treatment causes a decrease in the TBARS levels in skeletal muscle mitochondria of *mdx* mice. Moreover, we observed a significant decrease in the level of TBARS in the mitochondria of skeletal muscles of wild-type mice treated with MKT077.

**Figure 4:**
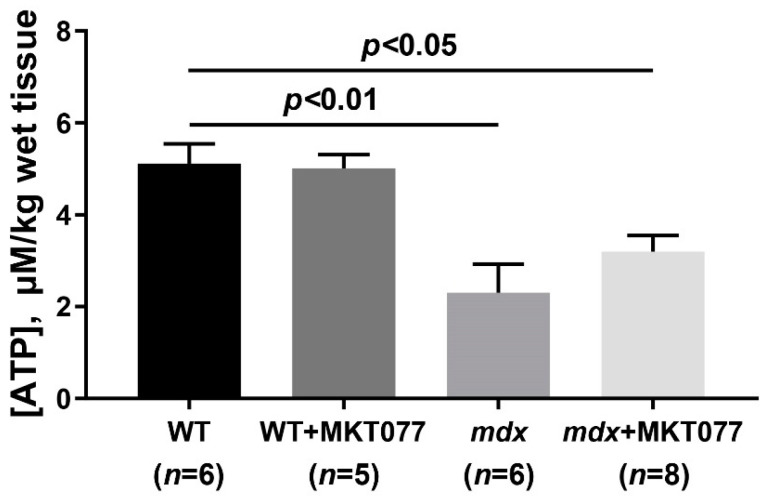
The effect of MKT077 on ATP levels in the quadriceps of mice. The data are presented as means ± SEM.

DMD is known to be associated with significant dysfunction of the OXPHOS system in skeletal muscle mitochondria and the suppression of ATP production required for muscle function [4]. As shown previously [9,11,24] and confirmed here (Table 1 and Figure S1), skeletal muscle mitochondria from *mdx* mice are characterized by a decrease in the respiration rate in the phosphorylating state (State 3), as well as the suppression of respiration in the presence of the protonophore uncoupler 2,4-dinitrophenol (DNP, state 3U_DNP_), which allows one to estimate the maximum rate of electron transport along the mitochondrial respiratory chain. This is also accompanied by a decrease in the respiratory control ratio (RCR), reflecting the coupling of respiration and phosphorylation in mitochondria. This suppression of OXPHOS efficiency may also result in decreased ATP levels in the skeletal muscle of *mdx* mice (Figure 4). One can see that MKT077 has no effect on the parameters of oxygen consumption by the skeletal muscle mitochondria of *mdx* mice, but reduces the efficiency of OXPHOS in the skeletal muscle mitochondria of wild-type mice. In particular, we noted a decrease in the respiratory rate in states 3 and 3U_DNP_, as well as the respiratory control ratio in the mitochondria of WT+MKT077 mice. At the same time, MKT077 has no effect on the ATP content in the skeletal muscles of both groups of mice. However, it can be assumed that, in this case, differences in the ATP level in the skeletal muscles of mice do not depend on the functional activity of mitochondria and, as previously suggested [25], are due to a decrease in muscle function in *mdx* mice, leading to a reduction in the basal level of ATP in the tissue. In addition, the activity of the respiratory chain under in vitro conditions is apparently excessive and this does not allow it to be adequately compared with the ATP level in the skeletal muscle of animals.

### 2.2. MKT077 Improves Skeletal Muscle Ultrastructure in mdx Mice but Causes Impairments in WT Animals

**Figure 5:**
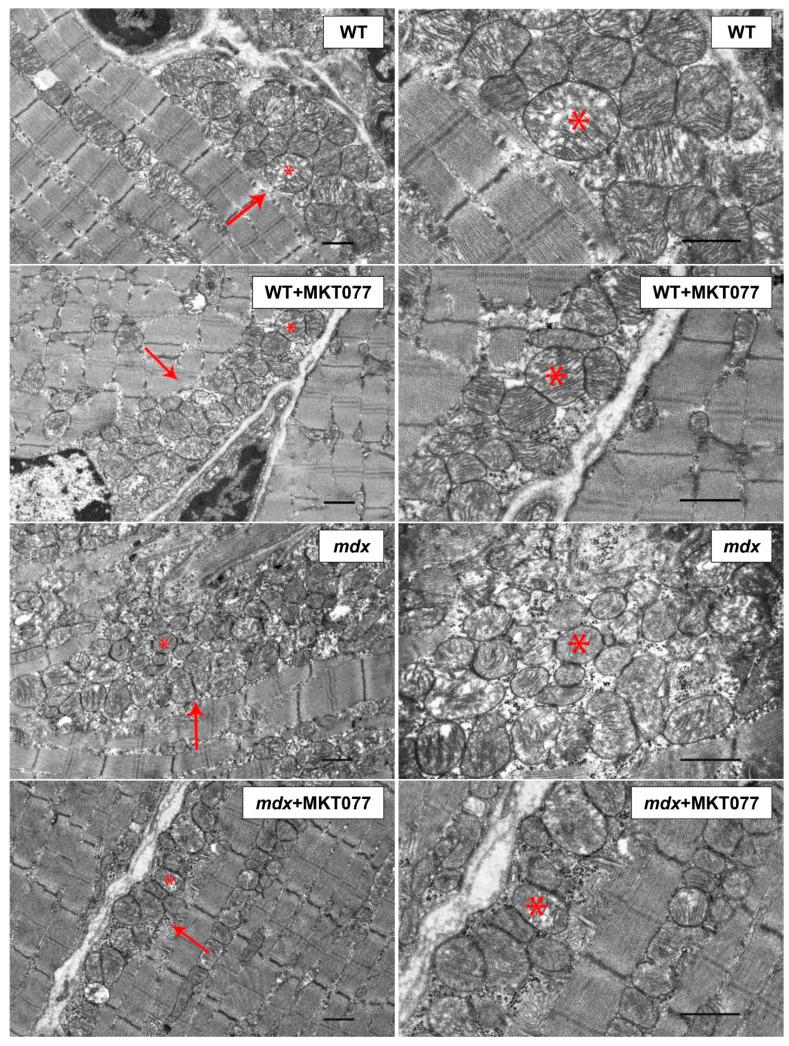
Representative transmission electron micrographs of mouse quadriceps sections. Mitochondria of the subsarcolemmal population are highlighted with red arrows. The asterisk marks the same mitochondria at low (**left side**) and high (**right side**) magnifications. Scale bar is 1 μm.

Dystrophin-deficient muscles are characterized by significant disruption of the ultrastructure, including the mitochondrial population [9,11,24]. This can be seen from the data in Figure 5. Skeletal muscle myofibrils from WT mice are characterized by normal M–Z band architecture. The band pattern appeared clear, the Z-lines were at right angles to the longitudinal axis of the myofibrils, and the myofilaments are well aligned with each other. In the subsarcolemmal region, located on the periphery of the muscle fiber, mitochondria form large clusters. Organelles have a spherical or elongated shape. There are no disturbances in the arrangement of the cristae or damage to the outer and inner membranes. The SR is mainly represented by small, flattened cisterns.

**Figure 6:**
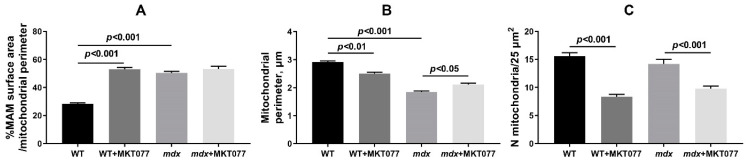
Electron micrograph (Figure 5) profiles: percentage of MAM surface area per mitochondrion perimeter in each microscopic field (**A**); mitochondrial perimeter (**B**) and number of mitochondria per plate (**C**). The data are presented as means ± SEM (*n* = 3).

The *mdx* group of mice exhibit altered sarcomere organization. In particular, Z-lines are often located obliquely relative to the longitudinal axis of the fiber or wavy across the fiber. Sometimes there are breaks in the Z-line. Myofilaments lose their precise alignment with each other due to the rupture of myofibrils. More often, a thinning of the myofibril diameter is observed without disruption of the organization of sarcomeres. Destructive changes also affect the sarcoplasm, SR, and mitochondria. The spaces of sarcoplasm between myofibrils are greatly expanded. In the sarcoplasm, we noted an increase in the contents of glycogen, secondary lysosomes, as well as strong proliferation and expansion of the cisterns of the SR, which surrounded the mitochondria on all sides. This is also accompanied by an increase in the area of SR/mitochondria contact sites (Figure 6A), consistent with our previous data [9]. In the subsarcolemmal region, mitochondria are located in abundant clusters. In these areas, attention is drawn to the enlarged, swollen and extended cisterns of the SR, which often occupy a large proportion of the subsarcolemmal space. The mitochondria are predominantly spherical in shape and have a much smaller diameter than those in the WT group (Figure 6B). Among the mass of normal mitochondria, there are mitochondria with destroyed cristae and zones of sharp enlightenment of the matrix. Often, cracks and ruptures are observed in the outer membrane of some of them.

MKT077 had no significant effect on skeletal muscle ultrastructure in *mdx* mice. At the same time, morphologically, the mitochondria of animals of the *mdx*+MKT077 group differ from the mitochondria of dystrophin-deficient animals by a denser and darker matrix, which brought them closer in structure to the mitochondria of wild-type animals (Figure 5). Along with this, we noted a decrease in the number of subsarcolemmal mitochondria in animals of the *mdx*+MKT077 group (Figure 6C). The effect of MKT077 was most pronounced in wild-type animals. In particular, in the skeletal muscles of WT+MKT077 mice, we observed an expansion of the sarcoplasmic spaces between myofibrils, containing extended, swollen, and dilated SR cisterns. In this case, the interfibrillar mitochondria are surrounded on all sides by cisterns and swollen vesicles of the SR. The effect of MKT077 is also accompanied by an increase in the area of SR/mitochondria contact sites in the muscles of WT mice (Figure 6A). The number of mitochondria under the sarcolemma is also reduced (Figure 6C), with mitochondria arranged in two or even one row (Figure 5). We also noted a decrease in the size of organelles in animals of the WT+MKT077 group (Figure 6B).

**Figure 7:**
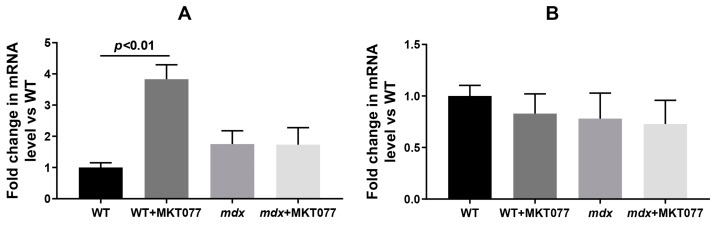
The relative mRNA expression of *Pink1* (**A**) and *Parkin* (**B**) relative to *Rplp2* in the skeletal muscles of experimental groups of mice. The data are presented as means ± SEM (*n* = 5).

One should note that the decrease in the number of mitochondria in the quadriceps of WT mice is accompanied by a significant increase in the expression of the *Pink1* gene (Figure 7A), which may indicate an increase in the intensity of mitophagy in the skeletal muscles of WT mice. At the same time, the expression of *Parkin*, encoding the E3 ubiquitin ligase recruited by Pink1 during the development of mitophagy, is not changed (Figure 7B). In the case of *mdx* mice quadriceps, changes in the expression of both *Pink1* and *Parkin* are not observed.

### 2.3. MKT077 Has No Effect on Increased SR Stress in Quadriceps of mdx Mice but Increases SR Stress in Muscles of WT Mice

**Figure 8:**
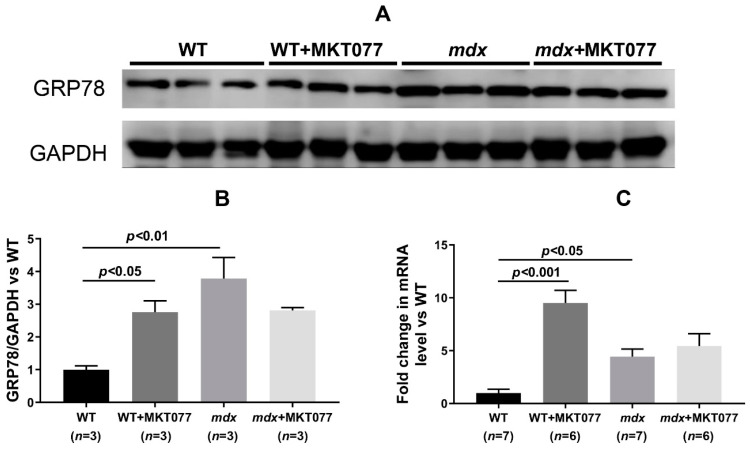
Western blotting of GRP78 and GAPDH (**A**), quantification of GRP78/GAPDH ratio (**B**) and mRNA expression of *Hspa5* relative to *Rplp2* (**C**) in the skeletal muscles of experimental groups of mice. The results are presented as means ± SEM.

It is known that modulation of proteins that form MAM contacts has a significant impact on SR function [16]. Dystrophin-deficient muscle fibers are characterized by significant dysfunction of the SR, which, in addition to disrupting the calcium-buffering capacity of this organelle, is also accompanied by the development of stress and stress-responsive activation of the unfolded protein response (UPR), limiting the accumulation of unfolded and misfolded proteins in SR [4,5]. Various SR stress and UPR markers are upregulated at different stages of dystrophin deficiency pathology development in *mdx* mouse muscles [26]. However, the most universal marker is the level of glucose-regulated protein 78 (GRP78) expression [26]. In our work, we noted both an increase in the GRP78 protein level and HSPA5 (GRP78) mRNA level in the quadriceps of 12-week-old *mdx* mice compared with wild-type animals (Figure 8), indicating the development of SR stress in the muscles of *mdx* mice. MKT077 treatment had no effect on the level of GRP78 protein and the expression of the *Hspa5* gene encoding this protein in the quadriceps of *mdx* mice, but significantly increased these parameters in wild-type mice, indicating the development of SR stress in WT+MKT077 animals.

### 2.4. MKT077 Reduces Skeletal Muscle Degeneration and Enhances Grip Strength of mdx Mice

**Figure 9:**
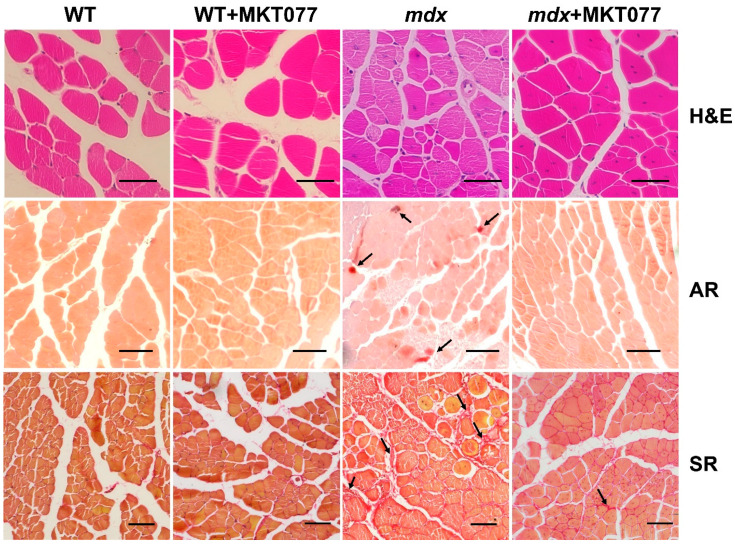
Representative histological images of the quadricep muscles of experimental mice: H&E staining, Alizarin red (AR) staining (calcified area indicated by arrows), and Sirius red (SR) staining (fibrotic area indicated by arrows). Scale bar is 75 μm (H&E and SR) or 150 μm (AR).

**Figure 10:**
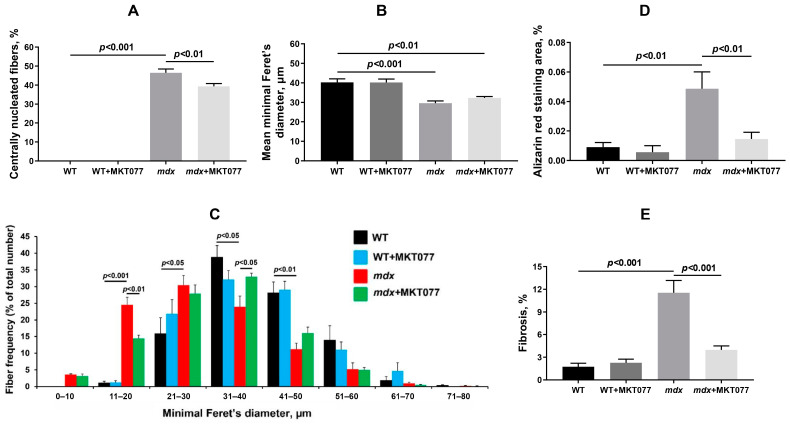
The percentage of CNF (**A**), mean minimal Feret’s diameter (**B**), fiber size distribution (**C**), percentage of the total fiber number), Alizarin red staining area (**D**), and the percentage of fibrosis (**E**) in the quadriceps of experimental animals. The data are presented as means ± SEM (*n* = 5).

In the next part of this work, we assessed the effect of MKT077 on the state of skeletal muscle tissue in mice, as well as the muscle strength and endurance of the animals. Figure 9 and Figure 10 show that H&E-stained quadricep samples of WT animals are characterized by the presence of peripherally nucleated fibers, since the majority of the muscle fiber is occupied by the contractile apparatus, and this is typical for healthy muscles. At the same time, dystrophin-deficient muscles, in particular the quadriceps of *mdx* mice, exhibit a significant increase in centrally nucleated fibers (CNFs), which have nuclei in the center of the cytoplasm. CNFs are recognized as regenerated myofibers and their levels reflect the intensity of the muscle fiber degeneration and regeneration cycles [27]. In addition, muscle fibers from *mdx* mice show a reduction in the mean minimal Feret’s diameter compared with WT mice. This is due to an increase in the proportion of small fibers (in the range from 0 to 30 μm) in the *mdx* mice muscles (Figure 10C), corresponding to newly formed muscle fibers during repeated cycles of regeneration and degeneration [28]. One can see that MKT077 significantly reduces the proportion of CNFs in the quadriceps of *mdx* mice (Figure 10A).

We did not detect an effect of MKT077 on the mean minimal Feret’s diameter of muscle fibers in *mdx* mice, but the distribution histogram data indicate a significant decrease in the proportion of small fibers 11–20 μm in size, as well as an increase in the proportion of fibers 31–40 μm in size in the muscles of *mdx*+MKT077 mice (Figure 10B,C), which, along with a decrease in the proportion of CNFs, also indicates a decrease in the intensity of degeneration/regeneration cycles of dystrophin-deficient muscle fibers.

Dystrophin-deficient muscles are characterized by the development of ectopic calcification caused by the deposition of calcium phosphate in the tissue [29]. Using Alizarin red staining, we also confirmed an increase in the level of tissue calcification in the quadriceps of *mdx*-mice (Figure 9 and Figure 10D). In this case, MKT077 contributed to the reduction of the Alizarin red staining area in the muscle of *mdx* mice to the level of control animals, which may indicate the elimination of ectopic calcification foci in the presence of this agent.

Another sign of degeneration of dystrophin-deficient muscle fibers is an increase in the level of fibrosis in skeletal muscles, indicating the replacement of functional muscle tissue with connective tissue elements. Indeed, Sirius red-stained quadricep samples of *mdx* mice show increased collagen deposition compared with WT animals (Figure 9 and Figure 10E). MKT077 causes a decrease in the proportion of fibrous tissue in the quadriceps of *mdx* mice to the level of wild-type animals. It is also important to note that fibrosis levels are also reduced in the diaphragm of *mdx* mice (Figure S2), which may reflect an overall beneficial effect of MKT077 on the dystrophin-deficient pathology.

**Figure 11:**
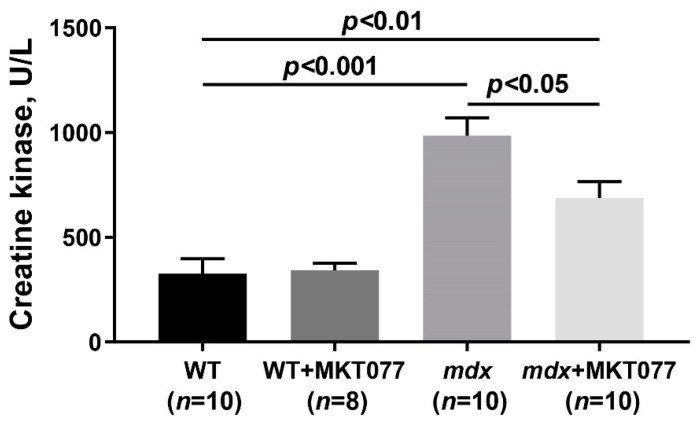
The effect of MKT077 on the activity of creatine kinase in the blood serum of mice. The data are presented as the mean ± SEM.

We also assessed the level of total creatine kinase in the blood serum of animals indirectly reflecting the intensity of damage to muscle fiber membranes. The level of this enzyme is significantly elevated in the serum of *mdx* mice compared with WT mice (Figure 11), which corresponds to the disruption of the membrane integrity of dystrophin-deficient fibers. In this case, MKT077 reduces the activity of creatine kinase in the blood serum of *mdx* mice, which may indicate the preservation of the integrity of the membranes of some muscle fibers of model animals.

**Figure 12:**
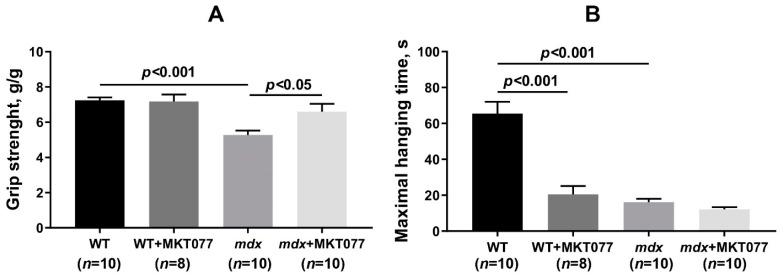
The effect of MKT077 on muscle strength and endurance in mice. (**A**) Grip strength test. (**B**) Wire-hanging test. The data are presented as means ± SEM.

Finally, we assessed the muscle strength and endurance of the tested groups of animals. For this purpose, we used grip strength and wire hanging tests. One can see that *mdx* mice show low levels of grip strength, as well as low wire hanging time compared with WT animals (Figure 12), which is consistent with previous data [9,11,24]. MKT077 causes a significant increase in the grip strength of *mdx* mice, but has no effect on the wire hanging time of animals. Moreover, MKT077 causes a significant decrease in the wire hanging time of WT mice, but does not affect the results of the grip strength test.

## 3. Discussion

One of the important consequences of Duchenne muscular dystrophy and the loss of dystrophin protein in striated muscles is the disruption of the integrity of the membranes of muscle fibers and cells (in the case of cardiomyocytes) and the dysregulation of ion homeostasis [4,5]. In particular, dystrophin-deficient muscles are characterized by a massive influx of calcium ions from the extracellular matrix leading to dramatic consequences, including the disruption of excitation–contraction coupling, disruption of muscle cell differentiation, proteolytic damage to cellular proteins by overactivated calpains, and disruption of the packaging of phospholipids in cell membranes and organelles by overactivated Ca^2+^-dependent phospholipases. These processes are accompanied by the development of oxidative stress, inflammation, and the replacement of muscle tissue with connective and adipose tissue (fibrosis) [2,4,5]. The administration of membrane sealants, such as Poloxamer 188 and its derivatives, as well as the suppression of the activity of membrane channels mediating excess calcium ion influx into the sarcoplasm, in most cases contributes to a significant mitigation of the pathological phenotype in various model dystrophin-deficient objects [30,31,32]. Another strategy to mitigate the effects of calcium homeostasis dysregulation in dystrophin-deficient muscles is to support the function of intracellular calcium depots, which are represented by the SR and mitochondria. In DMD conditions, these organelles are also in a state of chronic stress, which, in the case of SR, is expressed in a disruption of the calcium-storage function, control of secretory protein folding, lipid biosynthesis, and stress signaling, which, in particular, is accompanied by the activation of UPR, limiting the accumulation of unfolded and misfolded proteins [5]. The modulation of SR channels, for example, by activating SERCA, which is responsible for the absorption of calcium ions into the SR cisterns or, conversely, suppression of Ca^2+^ release from SR by blocking ryanodine receptor activity, contributes to the improvement of the disease phenotype [5].

Mitochondria also show significant dysfunction in dystrophin-deficient skeletal muscle, which may be due to secondary excess calcium ion supply from the sarcoplasm and calcium overload of the mitochondrial matrix (Figure 1), leading to the activation of the MPT pore, development of oxidative stress, and impairment of oxidative phosphorylation and ATP synthesis [4]. One of the strategies to improve mitochondrial calcium homeostasis and eliminate mitochondrial dysfunction is to block the induction of the pathological phenomenon of calcium-dependent MPT pores in the inner membrane of organelles. This can be achieved with specific MPT pore blockers, such as alisporivir or isoxazoles (like TR001), as shown in both animal models of DMD and patient biopsies [11,12,13,14,15]. This approach allows for significantly improving the state of skeletal muscle mitochondria and increasing their calcium-buffering function, which also alleviates skeletal muscle pathology, apparently due to the partial removal of excess calcium ions from the sarcoplasm. The strategy of suppressing MPT pore induction was also demonstrated in a *δ*-sarcoglycan-deficient mouse model, whose skeletal muscle mitochondria also exhibit calcium overload. In this case, knockout of proteins capable of forming the MPT pore channel, in particular, adenine nucleotide translocator (ANT), contributed to the improvement of the calcium-buffering function of mitochondria and significantly mitigated skeletal muscle pathology [10].

In the present work, using the most widely used dystrophin-deficient *mdx* mouse model, we examined another approach aimed at improving mitochondrial calcium ion homeostasis. For this purpose, we used rhodacyanine MKT077, which is capable of inhibiting the activity of the GRP75 protein directly involved in Ca^2+^ transfer from the SR to mitochondria and modulation of the functional activity of the latter [22,23]. One can see that the daily intraperitoneal administration of this agent to *mdx* mice at a concentration of 5 mg/kg for 28 days does indeed contribute to a reduction in the accumulation of calcium ions in the mitochondrial matrix of the skeletal muscles of the animals (Figure 1A). This is reflected in a reduction in calcium overload in freshly isolated quadriceps mitochondria from *mdx* mice and also evidenced by an increase in their ability to absorb external pulses of calcium ions (Figure 1B,C). One can see that the action of MKT077 is not accompanied by a change in the content of GRP75 protein in the skeletal muscles of mice and the level of the gene encoding this protein (Figure 2).

Excessive calcium overload of mitochondria is known to lead to the active production of ROS by organelles contributing to the development of mitochondrial dysfunction and oxidative stress, and also generally makes a significant contribution to the development of the pathology of dystrophin-deficient muscles [4]. The skeletal muscles of *mdx* mice are characterized by an increase in the level of lipid peroxidation products (MDA) in mitochondrial membranes (Figure 3), which can be considered as a marker of the development of oxidative stress [9,11,24]. In this case, MKT077, along with eliminating mitochondrial calcium overload, causes a significant decrease in the level of MDA in the organelles, which may indicate a decrease in the intensity of oxidative stress in treated dystrophin-deficient animals. This effect of MKT077 is not sufficient to reverse the reduced efficiency of oxidative phosphorylation in the skeletal muscle mitochondria of *mdx* mice (Table 1) and to influence the reduced tissue ATP levels (Figure 4). Moreover, in wild-type animals, MKT077 causes a significant suppression of the efficiency of oxidative phosphorylation in skeletal muscle mitochondria. One could assume that this effect of MKT077 in WT mice is also due to the suppression of calcium accumulation in the matrix of organelles, whose physiological concentration is an activator of mitochondrial enzymes, in particular, Krebs cycle dehydrogenases [7,8]. On the other hand, in this case, MKT077 also contributes to the reduction of MDA levels, which also confirms the general antioxidant effect of this agent. It should also be noted here that MKT077 has a possible inhibitory effect on the functional activity of the mitochondrial electron transport chain, previously shown via in vitro and in vivo experiments on rat liver mitochondria [33,34], which may both contribute to a decrease in the membrane potential in mitochondria and the production of smaller amounts of ROS, and a decrease in the efficiency of oxidative phosphorylation, which is most pronounced in the case of WT mice.

Electron microscopy data from mouse skeletal muscle (Figure 5) demonstrate that the effect of MKT077 is not sufficient to significantly improve the skeletal muscle ultrastructure of *mdx* mice, although the mitochondrial ultrastructure of these animals shows signs of improvement. Moreover, MKT077 administration leads to changes in the ultrastructure of skeletal muscles of wild-type mice, which was primarily expressed in the expansion of sarcoplasmic reticulum cisterns and an increase in the contact area of this organelle with mitochondria (Figure 6A). This response to MKT077 administration can be considered as an extensive adaptation of the skeletal muscles of WT mice to the disruption of Ca^2+^ transfer from SR to mitochondria, which is also observed in the case of skeletal muscles of control *mdx* mice, demonstrating a chronic complex disruption of calcium homeostasis [9]. Ultrastructural changes at the level of the sarcoplasmic reticulum in skeletal muscles of *mdx* mice and MKT077-treated WT mice are also accompanied by the development of stress of this organelle and SR stress-responsive UPR, the marker of which is an increase in the level of GRP78 protein (Figure 8), which, under SR stress conditions, dissociates from SR membrane proteins and migrates in the SR lumen to bind unfolded and misfolded proteins, preventing further disruption of homeostasis [26]. MKT077 has virtually no effect on the GRP78 level in the muscles of *mdx* mice, which may indicate the maintenance of high-intensity SR stress in the skeletal muscles of treated dystrophin-deficient animals. It should also be noted that there is a decrease in the number of mitochondria in the skeletal muscles of both groups of mice treated with MKT077 (Figure 6C). This, on the one hand, may be due to the suppression of calcium ion uptake into mitochondria, which plays an important role in the activation of mitochondrial biogenesis [35]. On the other hand, it cannot be excluded that the decrease in the number of mitochondria in muscles may be associated with the activation of the process of selective removal of defective mitochondria (mitophagy) in both groups of MKT077-treated mice. In particular, MKT077 is known to have anti-cancer activity, which is believed to be due to the selective induction of mitochondrial death in cancer cells [36,37]. An increase in the intensity of mitophagy can be indirectly indicated by a significant increase in the expression of the *Pink1* gene in the quadriceps of MKT077-treated WT mice (Figure 7A). 

Despite the absence of significant changes in the ultrastructure of skeletal muscles, we noted some positive effects of MKT077 on the function and histopathology of skeletal muscles in *mdx*-mice. We have noted a decrease in the area of ectopic calcification of skeletal muscles of MKT077-treated *mdx*-animals (Figure 9 and Figure 10D), which may be due, among other things, to the elimination of excess calcium overload of mitochondria (Figure 1). Indeed, it is known that mitochondria overloaded with calcium and, above all, calcium phosphate, can act as foci of ectopic calcification and mineralization of soft tissues [38]. This effect of MKT077 is accompanied by a decrease in the intensity of muscle fiber degeneration, as evidenced by a reduction in centrally nucleated fibers, a tendency toward the normalization of muscle fiber diameter, and a decrease in the level of creatine kinase in the blood serum of MKT077-treated *mdx* animals, reflecting the preservation of the integrity of the membranes of some muscle fibers (Figure 10 and Figure 11). Finally, we noted a decrease in the level of fibrosis in the skeletal muscles of MKT077-treated *mdx*-animals, which is known to often accompany the process of ectopic calcification of soft tissues [29]. Moreover, a reduction in fibrosis is observed not only in the quadriceps of *mdx* mice, but also in the diaphragm (Figure S2), which is known to be most susceptible to degenerative changes in this line of dystrophin-deficient mice exhibiting a relatively mild disease phenotype. The improvement of skeletal muscle state and preservation of muscle volume are also accompanied by an increase in indicators reflecting muscle strength in *mdx* mice. In particular, we noted an increase in the grip strength of MKT077-treated *mdx* mice to the level of wild-type animals (Figure 12A). At the same time, we did not detect any effect of MKT077 on the reduced wire hanging time of *mdx* mice (Figure 12B), which is more useful in assessing the endurance of animals. Moreover, MKT077 significantly reduces the wire hanging time of WT animals, which may indicate a decrease in the endurance of these animals. One could assume that the indicated negative effect is due to the known [33,34] and above-described MKT077-induced decrease in mitochondrial function, development of SR stress, and disturbances in the ultrastructure of skeletal muscles of WT mice. In *mdx* mice, these side effects of MKT077 may also prevent the significant normalization of muscle structure and function. Moreover, it can be logically assumed that the elimination of calcium overload of skeletal muscle mitochondria in *mdx* mice caused by MKT077 does not prevent, and perhaps even aggravates, the general calcium overload of the sarcoplasm of dystrophin-deficient muscle fibers that contributes to the pathogenesis of the disease. All this indicates a limited positive effect of MKT077 on the state of dystrophin-deficient skeletal muscles, but confirms the promise of modulating mitochondrial calcium homeostasis in the treatment of dystrophin-deficient skeletal muscle pathology.

## 4. Materials and Methods

### 4.1. Animals

Male C57BL10 mice (wild type, WT) and dystrophin-deficient *mdx* mice (C57BL/10ScSn-*mdx*) were from the Animal Breeding Facility, Branch of the Shemyakin and Ovchinnikov Institute of Bioorganic Chemistry, Russian Academy of Sciences, Russia (IBCh RAS Unique Research Device “Bio-model”, Pushchino, Russia). The following groups were used in this experiment: (1) wild-type mice treated with vehicle (WT, *n* = 10); (2) wild-type mice treated with MKT077 (WT+MKT077, *n* = 8); (3) *mdx* mice treated with vehicle (*mdx*, *n* = 10); and (4) *mdx* mice treated with MKT077 (*mdx*+MKT077, *n* = 10). Both mouse strains were treated starting at 8 weeks of age. 1-Ethyl-2-[[3-ethyl-5-(3-methyl-2(3H)-benzothiazolylidene)-4-oxo-2-thiazolidinylidene]methyl]-pyridinium chloride or MKT077 (formerly known as FJ-776) was from Chem Scene (Monmouth Junction, NJ, USA). MKT077 was dissolved in sterile saline and interperitoneally administered in doses of 150–200 μL (5 mg/kg per animal body weight) every day for up to 4 weeks, and control WT and *mdx* mice received sterile saline alone.

### 4.2. Grip Strength and Wire-Hanging Tests

The grip strength test (IITC Life Science, Woodland Hills, CA, USA) was used to assess the muscle strength of the animals. The results were presented in grams normalized to the body weight of the animal. Each mouse had three trials, and the average was used to evaluate the final result. 

The endurance of mice was assessed using a wire-hanging test. Each mouse was placed on a 3 mm string (38 cm long and 49 cm above a soft surface to cushion animals that fall off). The animal’s maximum wire-hanging time was used to assess endurance.

### 4.3. Creatine Kinase Assay

The creatine kinase activity in mouse blood serum was analyzed spectrophotometrically at a wavelength of 340 nm using a Multiscan Go plate spectrometer (Thermo Fisher Scientific, Waltham, MA, USA) and a commercially available test kit (Vector-Best, Novosibirsk, Russia).

### 4.4. Transmission Electron Microscopy

Quadriceps muscles (*vastus lateralis*, three samples per group from different animals) were processed as described previously [11,24]. To monitor the transverse orientation of muscle fibers and identify the mid-belly region of the muscle (according to [39]), semithin sections were first prepared and analyzed using an EVOS M5000 imaging system (Thermo Fisher Scientific). Ultrathin sections with a thickness of 60–70 nm were prepared from Epon blocks using a Leica EM UC6 ultramicrotome (Leica, Wetzlar, Germany). Visualization of the samples, which were stained with uranyl acetate and lead citrate, was carried out using a JEM-1400 electron microscope (JEOL, Tokyo, Japan) at the “Superresolution microscopy and spectroscopy” core facility at the Belozersky Institute of Physicochemical Biology. Electron microscopy images were analyzed using Image Tool 3.0 software. Morphometric analysis was performed using a routine method, requiring manual contouring of cross-sections of mitochondria along their outer membrane and membranes of the SR associated with mitochondria (MAM contacts) within 30 nm [40]. In total, 150 cross-sectional profiles of mitochondria (50 profiles/sample) were collected for each group of mice. Average values from each animal were used for analysis.

### 4.5. Histological Analysis

Quadriceps (*vastus lateralis*, five samples/group from different animals) and diaphragms (four samples/group from different animals) were embedded in paraffin after pre-treatment, as described previously [9]. Serial sections of 5 μm thickness were prepared using a Minux S710 rotary microtome (RWD, Shenzhen, China) and stained with hematoxylin and eosin (H&E) to assess the severity of histological changes, Alizarin red S to detect calcium deposits, and Sirius red to assess the level of fibrosis. Histological images (muscle mid-belly region identified according to [39]) were assessed using the EVOS M5000 imaging system (Thermo Fisher Scientific) and analyzed using ImageJ software version 1.53 (National Institutes of Health, Bethesda, MD, USA). The centrally nucleated fibers (CNFs) percentage and the minimal Feret’s diameter were calculated by counting all fibers in H&E-stained muscle sections. The area of calcified tissue was expressed as the percentage of tissue area stained with Alizarin red S to the total section area. The area of connective tissue (fibrosis) was expressed as the percentage of the area of tissue stained with Sirius red to the total area of the section. Average values from each animal were used for analysis.

### 4.6. Quantitative Real-Time PCR

Total RNA was prepared from 100 mg of deep-frozen quadriceps muscle samples (4–7 samples per group from different animals) using the ExtractRNA kit (#BC032, Evrogen, Moscow, Russia). For real-time PCR, the qPCRmix-HS SYBR reaction mixture (Eurogen, Moscow, Russia) and the DTLite5 amplifier (DNA-Technology LLC, Moscow, Russia) were used. Primer-BLAST was used for gene-specific primer selection and analysis (Table 2) [41]. The relative level of expression of each gene was normalized to the level of *Rplp2* mRNA, considered the most stable housekeeping gene in both healthy and injured skeletal muscles [42]. A comparative C_T_ method was used to assess the relative expression level of each gene [43].

### 4.7. Electrophoresis and Western Blotting

Ten milligrams of frozen quadriceps tissue were used to prepare total protein extract. To maintain integrity and function, complete protease inhibitor cocktail (P8340, Sigma-Aldrich, St. Louis, MO, USA), phosphatase inhibitor cocktail 3 (P0044 Sigma-Aldrich), Na_3_VO_4_ (1 mM), PMSF (1 mM), EDTA (1 mM), and EGTA (1 mM) were used. RIPA buffer (20–188, Merck Millipore Ltd., Billerica, MA, USA) was used for protein extraction. The Bradford assay (Bio-Rad Laboratories, Hercules, CA, USA) was used to quantify protein amount. The samples were diluted in Laemmli buffer, run on 12.5% SDS–PAGE (10 µg/lane), and transferred to a 0.45 µm nitrocellulose membrane (Cytiva, Marlborough, MA, USA). After overnight blocking, the membrane was incubated with the appropriate primary antibody. Anti-GRP78 BiP antibody (ab21685) was from Abcam (Cambridge, UK). Anti-GAPDH antibody (#AF0911) was from Affinity Biosciences (Cincinnati, OH, USA). Anti-GRP75 Antibody (#2816) was from Cell Signalling Technology, Inc. (Danvers, MA, USA). The corresponding secondary horseradish peroxidase-conjugated antibodies (7074, Cell Signaling technology Inc., (Danvers, MA, USA)) were used to detect immunoreactivity. Chemiluminescent ECL reagents (Pierce, Rockford, IL, USA) were used to determine the peroxidase activity. Proteins were visualized and quantified using the LI-COR system (LI-COR, Lincoln, NE, USA), LI-COR Image Studio software, and normalized to the GAPDH used as a loading control.

### 4.8. Isolation of Skeletal Muscle Mitochondria and Their Functional Analysis

The differential centrifugation method was used to isolate mitochondria from the total quadriceps of both hind limbs of mice [44]. The Bradford assay showed 20–30 mg mitochondrial protein/mL in the resulting suspension. The rate of O_2_ consumption by mitochondria was measured using the Oxygraph Plus system (Hansatech Instruments, King’s Lynn, UK) and incubation buffer contained 120 mM KCl, 5 mM NaH_2_PO_4_, and 10 mM HEPES-KOH (pH 7.4), and supplemented with 2.5 mM potassium malate + 2.5 mM potassium glutamate. Amounts of 0.3 mg mitochondrial protein/mL, 200 μM ADP, and 50 μM 2,4-dinitrophenol (DNP) were used in each assay. Typical polarographic curves are shown in Figure S1. The oxygen consumption rate was expressed in nmol O_2_/min/mg mitochondrial protein, and the respiratory control ratio as the ratio of the oxygen consumption rate in state 3 to the oxygen consumption rate in state 4 [45]. 

Ca^2+^ transport in mitochondria was assessed by calcium-sensitive indicator arsenazo III absorption using a Multiscan Go spectrometer at 675 and 685 nm (Thermo Fisher Scientific) [11]. To assess free matrix calcium, 2 mg mitochondrial protein/mL were resuspended in 210 mM mannitol, 70 mM sucrose, 1 mM KH_2_PO_4_, 10 μM EGTA, and 10 mM HEPES-KOH buffer (pH 7.4.), and supplemented with 50 μM arsenazo III. To induce non-specific permeabilization of mitochondrial membranes and calcium release, 0.1 mg/mL alamethicin (ALM) was used, which was accompanied by a sharp increase in arsenazo III absorption [9]. 

The mitochondrial Ca^2+^ retention capacity was estimated using the same system and incubation medium. For this purpose, calcium chloride was added in pulses of 20 μM until the spontaneous release of the ion from the mitochondrial matrix signaled the opening of the MPT pore in the inner membrane of the organelles.

Quantitative determination of thiobarbituric acid-reactive substances (TBARS, mainly malondialdehyde (MDA)) was used to express the intensity of mitochondrial lipid peroxidation.

### 4.9. Statistical Analysis

Results are expressed as means ± SEM. The statistical significance of the differences between the groups was evaluated using GraphPad Prism 8.0.1 and one-way analysis of variance (ANOVA), followed by the Tukey multiple-comparison post hoc test. A significance level of *p* < 0.05 was chosen for statistical significance.

## 5. Conclusions

The obtained results demonstrate once again that even partial normalization of calcium homeostasis, realized at the level of the sarcoplasmic membrane or intracellular organelles, can alleviate the pathology of dystrophin-deficient skeletal muscles. In this case, to achieve a significant effect on muscle function, it was sufficient to reduce Ca^2+^ transfer from SR to mitochondria at the level of MAM contacts, which prevented dramatic calcium overload of the mitochondrial matrix, leading to the activation of the MPT pore and the development of oxidative stress in these organelles. Thus, it can be concluded that the mitochondrial approach associated with reducing the calcium overload of the matrix of these organelles can be used for secondary therapy of Duchenne muscular dystrophy, but only as part of a complex therapy of this disease, affecting both the underlying cause (mutation in the gene and the absence of the dystrophin protein) and numerous secondary manifestations of the pathology.

## Figures and Tables

**Table 1:** Parameters of respiration and oxidative phosphorylation of mouse skeletal muscle mitochondria.

Group	V Respiration, nmol O_2_/min per 1 mg of Protein				RCR (Relative Units)
	State 2	State 3	State 4	State 3U_DNP_	
WT (*n* = 6)	32.8 ± 1.9	204.0 ± 3.0	33.3 ± 2.2	276.7 ± 8.6	6.0 ± 0.2
WT+MKT077 (*n* = 4)	31.1 ± 1.8	159.5 ± 6.8 *	33.3 ± 2.2	209.7 ± 11.4 *	4.8 ± 0.2 *
*mdx* (*n* = 6)	32.2 ± 2.1	175.0 ± 7.2 *	35.8 ± 2.5	229.5 ± 13.5 *	5.0 ± 0.2 *
*mdx*+MKT077 (*n* = 6)	28.8 ± 2.8	156.2 ± 9.1 *	32.7 ± 3.3	210.6 ± 10.9 *	4.9 ± 0.2 *

Respiration of mitochondria was fueled by 2.5 mM glutamate + 2.5 mM malate. Respiration of mitochondria in state 3 was initiated by 200 μM ADP. Mitochondrial respiration in the 3U_DNP_ state was initiated by 50 µM DNP. The data are presented as means ± SEM. * *p* < 0.05 versus WT group.

**Table 2:** List of gene-specific primers for RT-PCR analysis.

Gene	Forward (5′→3′)	Reverse (5′→3′)
*Hspa5*	TGAAGAGCTGAACATGGACC	CTCATCGGGGTTTATGCCAC
*Hspa9*	GACAAGGATGCCCAAGGTTC	GTAAAGACGCCTCCCAGAGT
*Pink1*	TTGCCCCACACCCTAACATC	GCAGGGTACAGGGGTAGTTCT
*Parkin*	AGCCAGAGGTCCAGCAGTTA	GAGGGTTGCTTGTTTGCAGG
*Rplp2*	CGGCTCAACAAGGTCATCAGTGA	AGCAGAAACAGCCACAGCCCCAC

## Data Availability

The data presented in this study are available upon request from the corresponding author.

## Supplementary Materials

The following supporting information can be downloaded at: https://www.mdpi.com/article/10.3390/ijms25189892/s1.
