# Supplementary material for: Reduction of Mitochondrial Calcium Overload via MKT077-Induced Inhibition of Glucose-Regulated Protein 75 Alleviates Skeletal Muscle Pathology in Dystrophin-Deficient mdx Mice
Source: Int J Mol Sci. 2024 Sep 13;25(18):9892. doi: 10.3390/ijms25189892 (PMC11432509; doi:10.3390/ijms25189892)
Supplement: Supplementary file 1 [file ijms-25-09892-s001.zip › ijms-3142081-supplementary.pdf]

International Journal of *Molecular Sciences*

MDPI

Article

Supplementary Material: Reduction of Mitochondrial Calcium Overload via MKT077-Induced Inhibition of Glucose-Regulated Protein 75 Alleviates Skeletal Muscle Pathology in Dystrophin-Deficient *mdx* Mice

Mikhail V. Dubinin [1,*], Anastasia E. Stepanova [1], Irina B. Mikheeva [2], Anastasia D. Igoshkina [1], Alena A. Cherepanova [1], Eugeny Yu. Talanov [2], Ekaterina I. Khoroshavina [1] and Konstantin N. Belosludtsev [1,2]

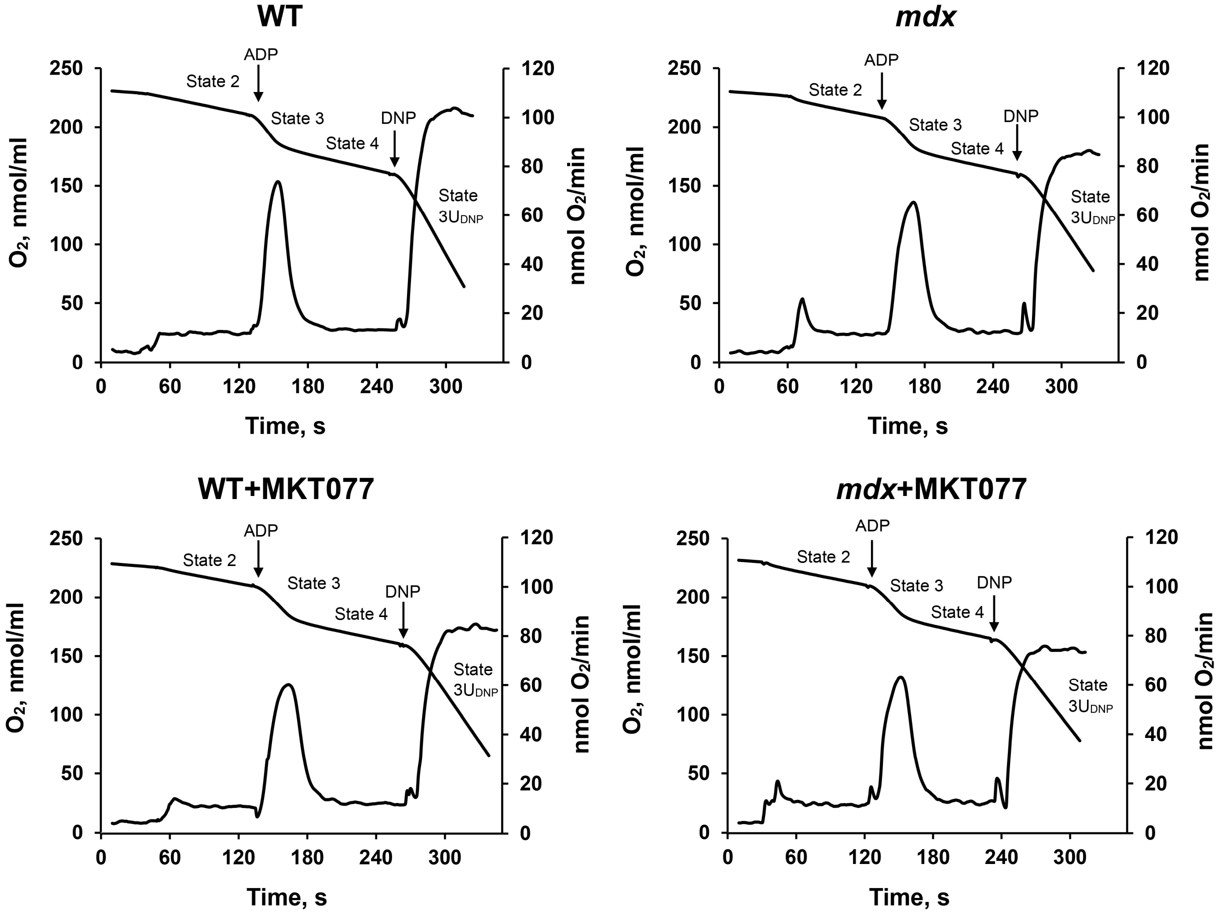

Figure S1. Typical curves of oxygen consumption by skeletal muscle mitochondria fueled by glutamate+malate and oxygen consumption rates. Medium composition: 120 mM KCl, 5 mM NaH_2PO_4, 2.5 mM potassium malate, 2.5 mM potassium glutamate, and 10 mM HEPES-KOH (pH 7.4). Additions: 200 μM ADP, 50 μM DNP.

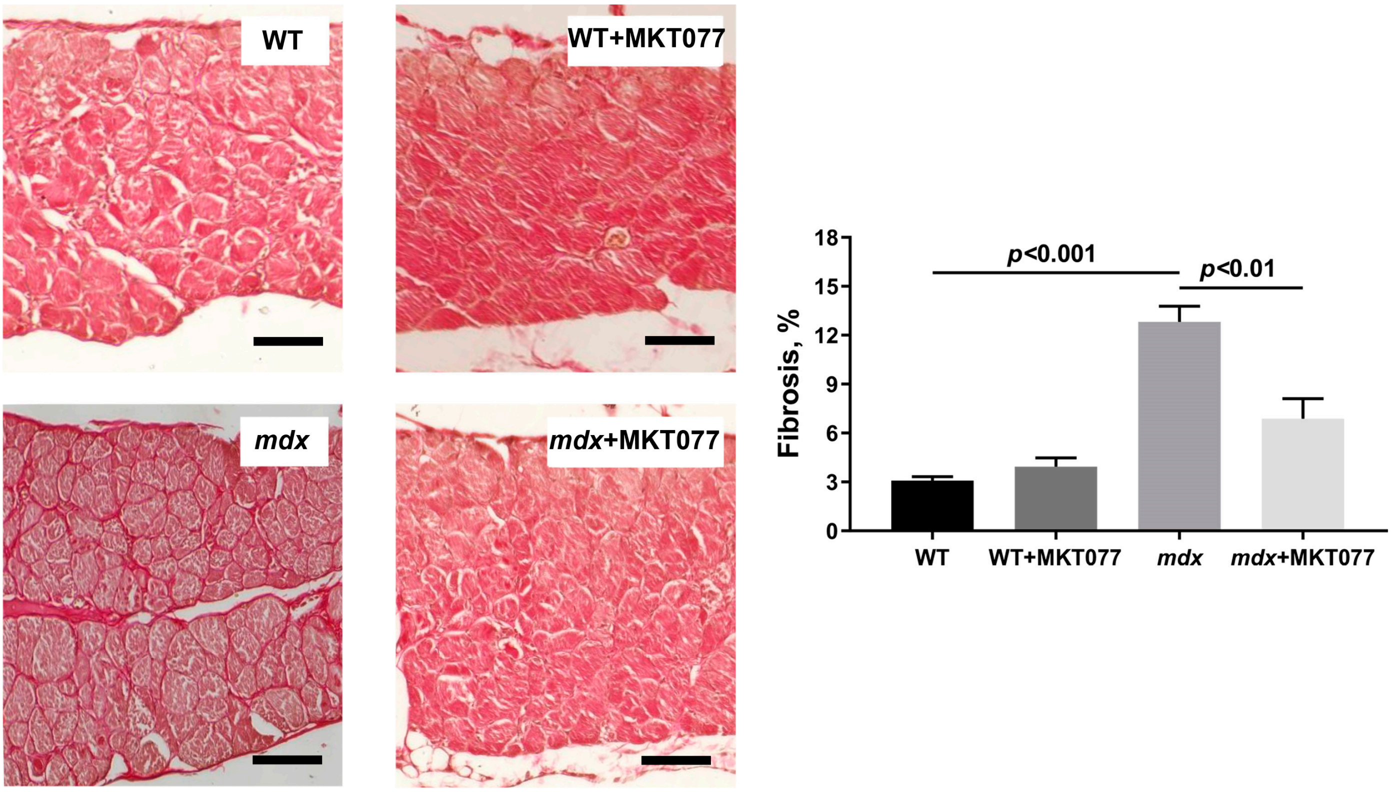

Figure S2. Representative histology images of diaphragms (Sirius red staining) and the percentage of fibrosis in the diaphragms of experimental animals. Scale bar is 75 μm. The data are presented as mean ± SEM (n = 4).
